# Highly efficient, *In-vivo* Fas-mediated Apoptosis of B-cell Lymphoma by Hexameric CTLA4-FasL

**DOI:** 10.1186/s13045-014-0064-6

**Published:** 2014-09-17

**Authors:** Alexandra Aronin, Shira Amsili, Tatyana B Prigozhina, Kobi Tzdaka, Roy Shen, Leonid Grinmann, Fanny Szafer, Per Edebrink, Mari-Anne Rauvola, Noam Shani, Michal Dranitzki Elhalel

**Affiliations:** Nephrology and Hypertension Services, Hadassah-Hebrew University Medical Center, Jerusalem, 91120 Israel; KAHR Medical LTD, Jerusalem, Israel; Cobra Biologics, Södertälje, Sweden

## Abstract

Non-Hodgkin lymphomas (NHLs) account for 4% of all malignancies. 5-year survival rate increased to 50% with new treatment modalities, however there is need for new effective treatment for the more aggressive, relapsing forms. Recently, CTLA4-FasL, that can bind to B7 and Fas receptor (Fas), was shown to induce robust apoptosis of cell lines originating from B cell lymphomas expressing both B7 and Fas, by activating pro-apoptotic signals in parallel to abrogating anti-apoptotic ones. The present study focuses on the unique properties of CTLA4-FasL as a potent apoptosis inducer of malignant cells *in-vitro* and in a xenograft model. CTLA4-FasL was found to naturally form a stable homo-hexamer. CTLA4-FasL induces robust apoptosis of a large variety of malignant cells while relatively sparing non-malignant ones, being more efficient when both receptors (B7 and Fas) are expressed on target cells. Even in non-B7 expressing cells, CTLA4-FasL exhibited better apoptotic activity than its parts, alone or in combination, however, only in B7 expressing cells apoptosis occurs at low concentrations and CTLA4-FasL induces activation of apoptotic signals and reduces anti-apoptotic ones. Importantly, CTLA4-FasL efficiently inhibited the growth of human B cell lineage tumors in a xenograft model, by provoking tumor cells’ apoptosis. Thus, CTLA4-FasL, a natural homo-hexamer protein, induces robust apoptosis of malignant cells, *in-vitro* and *in-vivo*. In B-cell lymphoma, its potency stems from the combination of its synergistic effect of activating the caspases while abrogating the anti-apoptotic signaling, with its unique hexameric structure, making CTLA4-FasL a promising candidate for aggressive B cell lymphomas treatment.

**Electronic supplementary material:**

The online version of this article (doi:10.1186/s13045-014-0064-6) contains supplementary material, which is available to authorized users.

## Introduction

Non-Hodgkin lymphomas (NHLs), as a disease set, is among the ten most malignant tumors, accounting for approximately 4% of all malignancies in both men and women [1]. NHLs are of B or T-lymphocytes lineage with most (80-90%) of them being of B-cell origin [2,3]. Though prognosis and treatment depend on specific type and stage, irradiation and chemotherapy have been proven effective in many NHL patients. New protein-based therapeutics, such as anti-CD20, have been recently added to the treatment toolbox [4]. The overall 5-year survival rate has increased to approximately 50%, but there is still need for new effective treatment for the more aggressive and relapsing forms of the disease [5]. Activated B-cells are known to express high levels of B7 receptors, also known as CD80 (B7.1) and CD86 (B7.2), which are required for T-cell activation as part of a co-stimulatory signal between the T-cell CD28 receptor and the B7 receptors on antigen-presenting cells including B lymphocytes [6]. Similarly to activated B-lymphocytes, B-cell lymphoma cells also express high levels of B7 molecules [7]. CTLA4 (Cytotoxic T-Lymphocyte Antigen 4), also known as CD152, is a Type-I membrane protein that down-regulates the immune response. CTLA4 is similar to CD28 in that they both bind to B7, however, whereas CD28 transmits a positive T-cell activation stimulatory signal, CTLA-4 does not. The membrane-bound CTLA-4 is known to function as a homodimer, interconnected by a disulfide bond [6]. CTLA4’s strong binding affinity to B7 led to the design of protein-based therapeutics, linking the CTLA4 extracellular domain to an antidody Fc domain (CTLA4-Fc), that is already approved for use in autoimmune diseases and transplantation [8]. In these chimeric constructs, both the CTLA4 and the Fc domains form a natural homo-dimer [8]. FasL is a Type-II membrane protein that naturally binds and activates Fas-receptors (Fas), resulting in cellular apoptosis [9,10]. FasL and Fas belong to the tumor necrosis factor (TNF) family. FasL:Fas interactions play a cardinal role in immune response modulation, as well as tumor growth and progression [11]. FasL, like other TNF super-family members, functions as a homo-trimer that binds to and signals through a trimerized Fas [12,13]_._ Upon FasL trimer binding and trimerization of Fas, a death-inducing signaling complex (DISC) is formed within the target cell, followed by activation of the caspases cascade and subsequent apoptosis [14]. Importantly, studies have shown that two adjacent trimeric FasL are required for optimal Fas signaling and the formation of DISC [15]. Also, hexameric recombinant form of FasL, termed as MegaFasL, was shown to induce robust, caspase-dependent apoptosis in Fas bearing cells [16,17]. Morover, hexameric FasL was found to recruit more Fas into lipid rafts than the trimeric form of FasL, in agreement with its higher efficacy [18].

Signal-Converting-Proteins (SCP) are a novel type of bi-functional fusion proteins that are formed by directly linking an extracellular domain of a type I membrane protein (extracellular amino-terminus), to the extracellular domain of a type II membrane protein (extracellular carboxyl-terminus), creating a fusion protein with two active sides. CTLA4-FasL is one such SCP, in which the N-terminal side is the extracellular domain of CTLA-4 and the C-terminal side is composed of the extracellular domain of Fas-ligand (FasL) [19]. Since CTLA4-FasL has the ability to bind to B7 molecules and to Fas, and in doing so, concurrently, to inhibit co-stimulation and induce apoptosis. CTLA4-FasL has been shown to efficiently induce apoptosis of activated T-cells [20] and to function as a strong immunomodulator in multiple autoimmune and transplantation animal models [21]. Recently, we have shown that CTLA4-FasL can induce robust apoptosis of B cell lymphoma cell lines by activating pro-apoptotic signals in parallel to abrogating anti-apoptotic ones [22].

The first study describing CTLA4-FasL identified it as a homo-trimer, but since CTLA4 naturally forms a homo-dimer, while FasL naturally forms a homo-trimer, the authors raised the possibility that CTLA4-FasL can form a homo-hexamer on the surface of the target cell when anchored to the B7 molecules through the CTLA4 moiety [19]. In the present study, we present data suggesting that CTLA4-FasL naturally forms a stable and soluble homo-hexamer and propose a unique mode-of-action model for the treatment of B cell lymphoma. We also demonstrate that CTLA4-FasL confers a specific and highly effective killing of tumor cells of the B cell lineage, both *in-vitro* and *in-vivo*, indicating that CTLA4-FasL might have a possible role as a new anti-cancer agent.

## Results

### CTLA4-FasL purification

**Figure 1:**
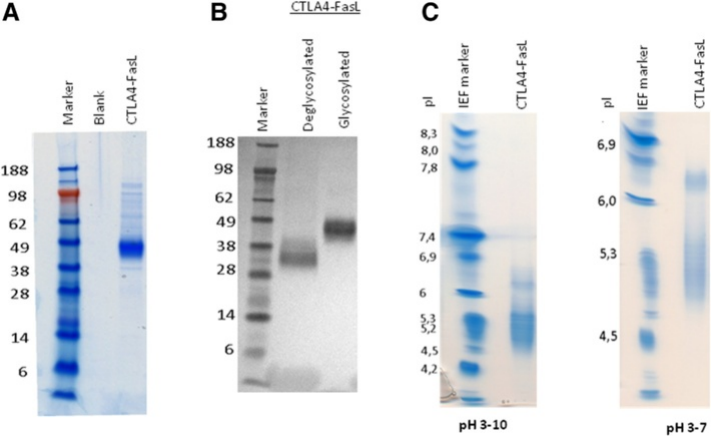
**CTLA4-FasL molecular structure. (A)** SDS-PAGE analysis of the CTLA4-FasL under reducing conditions, coomassie G-250 stain. **(B)** Western blot analysis using anti CTLA4 antibody following enzymatic removal of the N-glycan chains from the protein. **(C)** Iso-Electric Focusing analysis of CTLA4-FasL at pH3-7 and pH3-10.

To start, we looked at the purified CTLA4-FasL using SDS-PAGE. Although the predicted molecular weight of CTLA4-FasL is approximately 31kD, the fusion protein migrates in reduced conditions as a protein of approximately 43kD (Figure 1A). Treating the production media samples with the “Peptide N-Glycosidase F” enzyme that removes N-glycan chains from the protein, resulted in a shift in molecular weight (MW) from ~45 kDa to ~33 kDa (Figure 1B), indicating that the apparent difference in MW is due to protein glycosylation. In addition, by using Iso-Electric-Focusing analysis, we found that the actual iso-electric point (pI) of CTLA4-FasL is approximately 4.7-5.2, while its theoretical pI is 6.59, supporting the notion that CTLA4-FasL is glycosylated (Figure 1C).

As mentioned in the material and methods section, utilizing the glycosylation of CTLA4-FasL, a preliminary purification process was developed, in which Concanavalin-A (Con-A) chromatography was used as the main capture step. This was followed by two successive size-exclusion chromatography (SEC), yielding CTLA4-FasL at over 90% purity as measured by SDS-PAGE (Figure 1A).

### CTLA4-FasL forms a hexamer

**Figure 2:**
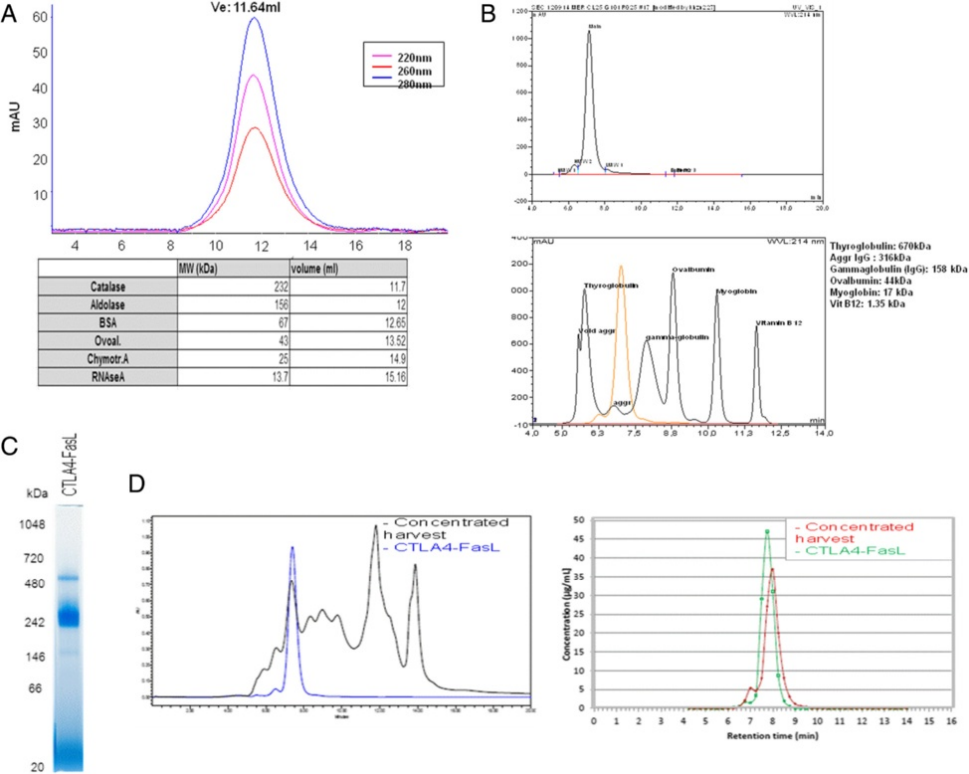
**Higher order structure of the CTLA4-FasL. (A)** Gel filtration chromatography. The retention volumes of the reference proteins are presented in the table below. **(B)** Analytical SEC-HPLC. The retention times of the reference proteins are presented in the lower panel. **(C)** Native-PAGE analysis. **(D)** SEC-HPLC analysis of the concentrated CHO-S cells harvest (left panel, black, as compared to the purified CTLA4-FasL profile, blue), followed by a Gyrolab analysis of CTLA4-FasL content in the fractions (right panel, red dots, as compared to the purified CTLA4-FasL profile, green dots).

To further study the higher-order structure of CTLA4-FasL, purified CTLA4-FasL was initially analyzed by gel-filtration chromatography. The protein peak of CTLA4-FasL fractionated at a volume similar to that of Catalase, with MW of 232kD; indicating that most of the CTLA4-FasL protein migrates as a peak of approximately 250kD (Figure 2A). Since this observed product size of about 250kD was significantly larger than the predicted homo-trimer suggested previously (e.g., ~130kD) [19], analytical Size-Exclusion High-performance Liquid Chromatography (SE-HPLC) and native-PAGE were used to study the actual product size at higher resolution. Surprisingly, by using SE-HPLC we found that roughly 90% of the fusion protein migrates as a peak of approximately 250kD, which is consistent with the size of a homo-hexamer, while the rest of the protein (~5-10%) was found mostly as a higher-molecular-weight (HMW) peak (Figure 2B). When the samples were analyzed by Native-PAGE, an identical pattern was found (Figure 2C), with most of the protein migrating as a 250kD band and a minor band of approximately twice that size, i.e., 500kD.

To test if the CTLA4-FasL homo-hexamer structure is formed only after purification, at the highly concentrated preparations of the protein, a similar SE-HPLC analysis was performed on harvested production media, before any purification was carried out, and the amount of CTLA4-FasL in the SE-HPLC fractions was quantified by CTLA4-FasL Gyrolab analysis. As can be seen in Figure 2D, most of the CTLA4-FasL in the harvest media (based on Gyrolab analysis) corresponds to a large SE-HPLC peak with retention-time identical to that of the CTLA4-FasL homo-hexamer, suggesting that the vast majority of the CTLA4-FasL fusion protein is in the form of a homo-hexamer structure already at the concentrated harvest media, before any purification took place.

### CTLA4-FasL induced apoptosis is correlated with relevant receptors expression patterns on target cells

The unique structure of the CTLA4-FasL chimera, predicts Fas-related apoptotic activity coupled to B7 targeting. To assess if these indeed is the case, we first measured the *in-vitro* killing activity of purified CTLA4-FasL on 13 different malignant and non-malignant human cell-lines. CTLA4-FasL was found to induce a significant, dose dependent killing effect in seven out of the ten cancer cell-lines we assessed, while it had almost no killing effect on the three non-malignant lines tested (Table 1).

**Table 1:** **CTLA4-FasL or His**_**6**_ **-CTLA4-FasL cytotoxic effect on different malignant and non-malignant human cell-lines**

**Cell-line type**	**Cell-line**	**Killing effect**	**~EC50**	**Incubation time**
Human liver cancer	Hep-G2	Positive	1.0 nM	24 hours
	SK-Hep1	Positive	1.5 nM	24 hours
	Huh-7	Negative	>>120 nM	48 hours
Human liver cells (non-malignant)	FH-B	Negative	100 nM	24 hours
Human kidney cancer	A498	Positive	1.0 nM	24 hours
	Caki-1	Positive	2.0 nM	24 hours
	786-0	Positive	1.0 nM	24 hours
Human kidney cells (non-malignant)	PCS-400-010	Negative	100 nM	24 hours
	PCS-400-011	Negative	100 nM	24 hours
Human lymphoma (B cells)	Raji	Positive	0.02 nM	24 hours
	JY	Positive	0.04 nM	24 hours
Human multiple myeloma	RPMI 8226	Negative	>120 nM	24 hours
Human leukemia	HL-60	Negative	>>120 nM	24 hours

Different human cell lines were incubated with different concentrations of CTLA4-FasL for 24 or 48 h. Cell viability was tested using the MTS assay.

**Figure 3:**
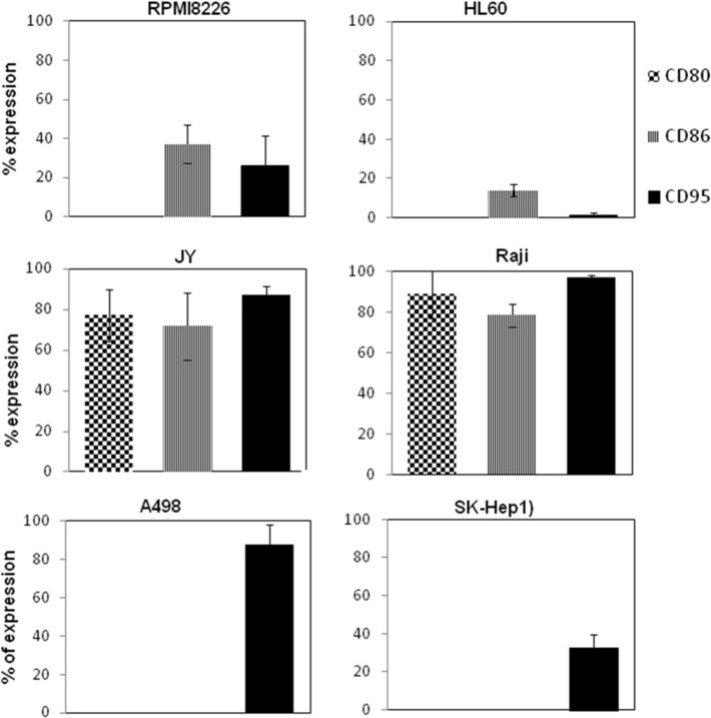
**Receptors expression on different human cell lines.** The protein expression level of B7-1 (CD80), B7-2 (CD86), and Fas (CD95) was determined by immunostaining of cells with the corresponding antibodies, followed by flow cytometeric analysis. The results represent the average of at least two independent experiments +/- SD.

As predicated, enhanced CTLA4-FasL killing effect was observed when human B cell lymphoma cancer cell-lines were cultured in the presence of the protein (Table 1). Of note, no viable cells of the B lineage could be detected in cultures were CTLA4-FasL was added at 30 ng/ml and above whereas in other, B7 negative cell lines this maximal effect of CTLA4-FasL could be seen only at concentrations above 300 ng/ml or not at all (Additional file 1: Figure S1 and not shown). This is of particular importance since these B-cells are known to express B7 receptors, suggesting a correlation between activity and specific receptor expression. To study this hypothesis, we used FACS analysis to quantify the expression of the three target receptors of CTLA4-FasL, namely CD80 (B7.1), CD86 (B7.2) and CD95 (Fas), on the different human cancer cell lines. As can be seen in Figure 3, the APL HL60 Human Leukemia cell line, found to be CTLA4-FasL resistant by the bioassay, expresses very low levels of surface CD86 and undetectable CD80 and Fas levels. Similarly, the multiple myeloma cell line, RPMI8226 also found to be CTLA4-FasL resistant, expresses only low surface levels of Fas and CD86, with no CD80. In contrast, the JY and Raji B cell lymphoma cell lines, shown to be highly sensitive to CTLA4-FasL, express high levels of CD80, CD86 and CD95 (Figure 3 and Additional file 1: Figure S1B). Cell lines expressing only Fas (A498 and SK-HEP1) were moderately sensitive to CTLA4-FasL. These findings suggest that cells expressing both receptors are highly sensitive to CTLA4-FasL, cells expressing just Fas, are moderately sensitive, while cells that express none of the receptors or just B7 are resistant to CTLA4-FasL’s effect. Importantly, we previously tested another fusion protein, CD40-FasL, that cannot bind to B7 molecules [22]. CD40-FasL was much less potent in inducing apoptosis of the B cell lineage cell lines expressing both B7 and Fas than CTLA4-FasL, but was extremely effecting in causing apoptosis of CD40L and Fas expressing cells. As we now find that CTLA4-FasL is a hexamer, we performed a gel filtration of the CD40-FasL conditioned medium to test whether it is also a natural hexamer. Gel filtration fractions were loaded on SDS-PAGE and subjected to Western blot analysis using anti FasL Ab (Additional file 2: Figure S2B) or analyzed by CD40 ELISA (Additional file 2: Figure S2C). CD40-FasL was found mainly in fractions corresponding to ~ 300 – 500 kDa indicating a hexameric structure. As both proteins are hexamers, the fact that CD40-FasL is extremely effective in inducing apoptosis in CD40L and Fas expressing cells [22], but has much lower activity on B7 and Fas expressing cells when compared to CTLA4-FasL, supports the importance of the CTLA4 binding to the B7 molecules for inducing the robust apoptotic effect of CTLA4-FasL on B7 expressing cells.

### CTLA4-FasL apoptosis-based effect is greater when compared to its two subunits or their combination

**Figure 4:**
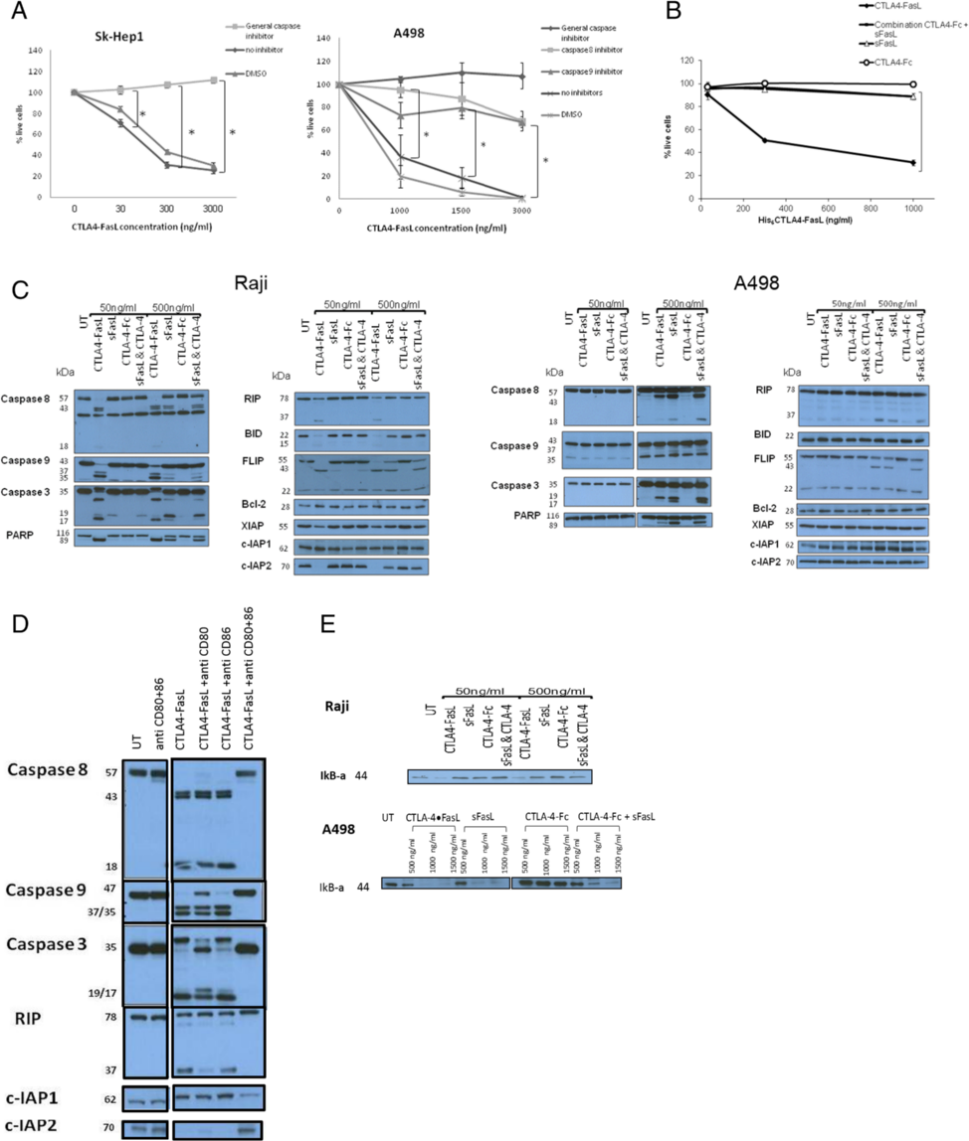
**CTLA4-FasL effect on pro and anti apoptotic signals. (A)** Sk-Hep1 (left) and A498 (right) cell lines were pre-incubated with or without caspase inhibitors (Z-VAD-FMK (general), Z-LEHD-FMK (caspase 9), Z-IETD-FMK (caspase 8)) for 1 hour followed by incubation with his_6_CTLA4-FasL at different concentrations for 24 hours. Cells’ viability was tested by the MTS assay. The results represent the average of three independent experiments. +/- SE (*p ≤ 0.05). **(B)** Sk-Hep1 cells were incubated with CTLA4-FasL, sFasL, CTLA4-Ig or combination of the later two for 24 hours. Cell viability was tested by the MTS assay. The results represent the average of four independent experiments. +/- SE (*p ≤ 0.05). **(C)** CTLA4-FasL effects on the expression of apoptotic and anti-apoptotic proteins in B cell lymphoma cell lines (left) and RCC (right). Raji and A498 cell lines were incubated with indicated concentrations of CTLA4-FasL, sFasL, CTLA4-Ig or the combination of the later two for 2 h. Whole cell lysates were analyzed by Western blot. These are representative results of the three independent experiments. **(D)** Effect of B7 blockade on CTLA-FasL’s effect on pro and anti apoptotic signals – Raji cell lines were incubated for 1 h with 1 μg/ml of anti CD80, anti CD86 or both prior to the addition of CTLA4-FasL (50 ng/ml). Cells were collected after 2 h. Whole cell lysates were analyzed by Western blot. **(E)** CTLA4-FasL effect on NFκB pathway - A498 (lower panel) and Raji (upper panel) cell lines were incubated with indicated concentrations of CTLA4-FasL, sFasL, CTLA4-Ig or the combination of the later two for 2 h (Raji) or 6 h (A498). Whole cell lysates were analyzed by Western blot.

We have shown in the past that his_6_-CTLA4-FasL induces efficient apoptosis of lymphatic cancer cells by utilizing a dual signaling pathway that includes Fas-mediated apoptosis of CD95 expressing cells, coupled to the abrogation of cFLIP expression in cells that express B7 as well [22]. Also, we have previously shown that CTLA4-FasL inhibitory effect on T lymphocytes activation is mediated by apoptosis induction, through the caspases cascade [20]. To further investigate CTLA4-FasL mode-of-action in cancer cell line, we studied if CTLA4-FasL cytotoxic effect can be abrogated by the pan-caspase inhibitor (Z-VAD), caspase 8 inhibitor (Z-IETD-FMK) and caspase 9 inhibitor (Z-LEHD-FMK) on malignant cell lines positive for Fas only. As can be seen in Figure 4A, the pan caspase-inhibitor resulted in full inhibition of CTLA4-FasL killing effect of the Sk-Hep1 and A498 cell lines. The inhibitors of caspase 8 and 9 resulted in partial inhibition, supporting the assumption that CTLA4-FasL activity is mediated by both the intrinsic and the extrinsic apoptotic pathways. Of note, caspase 8 inhibitor was more potent than the caspase 9 inhibitor.

SCP chimeras have been shown to confer superior activity over their parts, separately or in combination [19,22]. However, this was tested previously only in target cells that express binding molecules to both SCP sides [19,22]. As the hepatocellular carcinoma (HCC) cell lines SK-Hep1 and HEPG2, do not express B7 molecules (Figure 3 and not shown), and therefore can bind to the FasL only, we wanted to test if this superior activity will still be evident. For that, cells were incubated in the presence or absence of soluble CTLA4 (CTLA4-Fc), soluble FasL (FLAG-FasL) or the combination of the latter two for 24 h, and cell viability was measured by MTS. As seen in Figure 4B CTLA4-FasL’s cytotoxic effect is significantly more potent than that of its components, even when combined. Thus, CTLA4-FasL is superior to its components even in non-B7 expressing cells, suggesting its FasL domain is presented to the Fas in an exceptionally effective way, probably because it is a hexamer and not a trimer, as it was previously shown that a hexameric FasL is more potent than a trimeric one [15,23].

Next, we studied the effect of CTLA-FasL and its separate parts on the FasL: Fas signaling cascades in cells that either bear or lack B7. As can be seen in Figure 4C, in the A498 cell line, that is devoid of B7 expression, CTLA4-FasL induces effective propagation of the pro-apoptotic signaling such as caspase 3, 8 and PARP cleavage, only at high concentrations (500 ng/ml) and does not differ from sFasL [24]. In contrast, in Raji cells, that do express B7, the effect of CTLA4-FasL is already evident at 50 ng/ml and the superiority over sFasL is apparent. In addition, at the lower concentrations, the proapoptotic protein BID is truncated to its activated form tBID, and caspase 9 is cleaved only in the B7-expressing Raji cells (Figure 4C and Additional file 3: Figure S3A-H), again stressing the advantage of CTLA4-FasL, and imply the involvement of the mitochondrial apoptotic pathway [25,26]. Also, cFLIP-L that is known as an anti-apoptotic protein that interferes with caspase 8 activation is cleaved to its N-terminal form p43 [27,28]. We find rapid abrogation of FLIP-L expression at 50 ng/ml in CTLA4-FasL treated cells as opposed to sFasL treated cells only in B7 expressing cells. Importantly, in the B7-positive Raji cell line, we find a significant decrease in the expression of the anti-apoptotic protein c-IAP2 [29,30], while no such changes was seen in the A498 cells even at the higher concentration of CTLA4-FasL (Figure 4C). This effect was completely abrogated by using blocking antibodies against CD80 and CD86, stressing the importance of the binding of the CTLA4 domain to the B7 molecules (Figure 4D).

All of these observations suggest that CTLA4-FasL is more potent than sFasL at low concentrations in cells expressing both B7 and Fas. Similar findings were found with JY, another B7 expressing cell line of the B-cell lineage (data not shown). Of note, as activation of the Fas was shown to induce pro-proliferative signals [31], we looked at the expression of IkappaB-α. The expression of IkappaB-α is decreased in B7 negative cells treated with CTLA4-FasL from the concentration of 1000 ng/ml and above, and already at 50 ng/ml in B7 positive cells (Figure 4E).

### CTLA4-FasL inhibits tumor growth and improves mice survival in a B-cell lymphoma xenograft model

**Figure 5:**
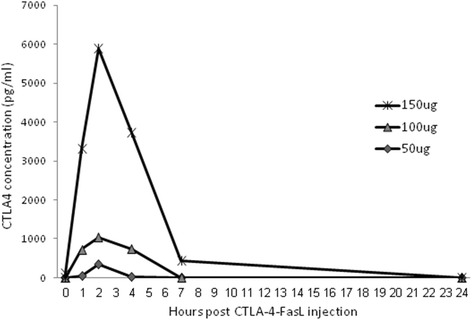
**Pharmacokinetic analysis of CTLA4-FasL.** CTLA4-FasL at different doses was injected s.c. to mice at a total volume of 150 μl per mouse. Mice were sacrificed at various time points (0-24 h) post injection. CTLA4-FasL level in plasma was quantified by Human Soluble CTLA-4 ELISA kit.

Prior to initiation of studies in a mouse disease model, we measured the basic pharmacokinetic (PK) parameters of CTLA4-FasL in mice. The protein serum levels were quantified by a CTLA4 commercial ELISA at specific time points following subcutaneous (sc) injections. CTLA4-FasL levels were shown to reach the highest values approximately 2 hours post injection with T1/2 of approximately 4-5 hours post injection (Figure 5). Similar results were obtained in both Balb/C and NUDE mice (not shown).

**Figure 6:**
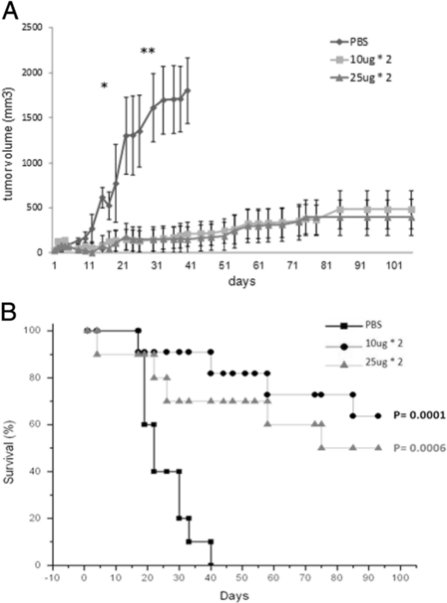
**CTLA4-FasL inhibits tumor growth** ***in vivo*** **.** Ex vivo JY cells were injected subcutaneously to irradiated NUDE mice. At day 5 after JY injection, tumor volume was calculated and mice were treated daily with subcutaneous injections of CTLA4-FasL or PBS for 5 days. **(A)** Tumor volume. Results represent the mean +/- SE volume of tumors, (n = 10 for each group). *P < 0.05 between PBS group and 10 ug*2 or PBS vs 25 ug*2. **P <0.01 respectively. **(B)** The survival curve of the experimental groups and control.

**Figure 7:**
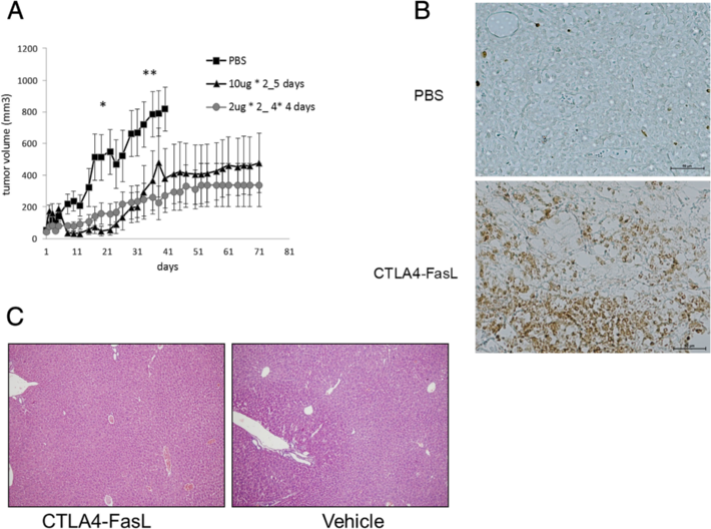
**CTLA-4•FasL inhibits tumor growth** ***in vivo*** **.** **(A)** Ex vivo JY cells were injected subcutaneously to irradiated NUDE mice. At day 5 after JY injection, tumor volume was calculated and mice were treated daily with subcutaneous injections of CTLA4-FasL (10 μg twice a day for 5 days for one treatment course or 2 μg twice a day for 4 days for four consecutive weeks). Control animals received similar volume of PBS. The results represent the mean +/- SE volume of tumors (n = 10 for each group). * - P <0.05, **P <0.01 between PBS group and treatment group **(B)** Five days post s.c. injection of human JY tumor cells to irradiated-NUDE mice the mice were treated with 10 ug CTLA4-FasL or PBS twice a day for 5 consecutive days. Tumors were harvested one hour after last injection, fixated and embedded in paraffin, and tissue sections were processed and stained with anti cleaved caspase 3 antibody. **(C)** 8 days following last CTLA4-FasL (100 μg a day, 4 days), or vehicle injection mice livers were harvested, fixated and embedded in paraffin, and stained with H&E.

For exploring CTLA4-FasL efficacy *in-vivo*, NUDE mice were injected (sc) with JY cells and followed daily for tumor growth. When tumors were palpable they were treated based on the PK results, with twice-daily sc injections of various CTLA4-FasL dosages or vehicle for 4 consecutive days. As illustrated in Figure 6, treatment with both 50 ug and 20 ug daily dosages of CTLA4-FasL for 4 days, significantly inhibited the growth of human JY xenograft tumors (Figure 6A) and significantly improved survival of the treated mice (Figure 6B). Since the 20 ug/day dose was found to be as effective as the 50ug dose, we next tested the effect of lower dosages. In a second experiment we found that five days administration of 10 ug/day significantly inhibited tumor growth, with a significant effect lasting to ~20 days, while a low dose treatment of 4 ug/day for 4 consecutive days, which was repeated for 4 weeks, seems to keep tumor volumes at a stable reduced state (Figure 7A).

In agreement with tumor volume and the survival indexes, the high efficacy of CTLA4-FasL treatment of JY xenograft tumors was further illustrated by the immunostaining of tumors removed from the mice, with anti-cleaved casapase 3. As seen in Figure 7B, almost all tumor cells in CTLA4-FasL treated mice undergo apoptosis, while only very few tumor cells from vehicle treated mice stained positive to anti cleaved caspase 3. As FasL and agonistic anti-Fas Abs were previously described to be significantly hepatotoxic [32,33], at another experiment mice from vehicle or 100 microgram/day CTLA4-FasL treated groups were sacrificed 8-30 days after last injection. Representative harvested livers stained with hematoxilin eosine are seen in Figure 7C. In Livers harvested from mice treated with 100 μcg a day for 4 days no significant liver damage was observed. Of note, at higher doses, liver toxicity was evident, especially when higher molecular weight forms of CTLA4-FasL as dodecamers were present (not shown).

## Discussion

In the present study we investigated the unique properties of the signal converter protein CTLA4-FasL as a potent apoptosis inducer of malignant cells. The main findings are: 1. CTLA4-FasL naturally forms a stable homo-hexamer; 2. CTLA4-FasL induces robust apoptosis of malignant cell lines while relatively sparing non-malignant ones; 3. The CTLA4-FasL killing effect is more efficient when both relevant receptors (e.g. B7 and Fas) are expressed on target cells; 4. Even in non-B7 expressing cells, CTLA4-FasL exhibited significantly higher apoptotic activity than its parts, alone or in combination; 5. In B7 expressing cell CTLA4-FasL is highly efficient in activating apoptotic signals while diminishing the anti-apoptotic ones, and 6. CTLA4-FasL efficiently inhibited the growth of human B cell lineage tumors in a xenograft model.

Bi-specific and multi-specific biological drugs are believed to develop into the “next generation” of protein-based drugs. Mostly combining functional units of two known biological targets, this drug-development field is currently lead by bi-specific antibodies [34,35], while other bi-specific technologies, such as Signal Converter Proteins, are being assessed as well [20,22,36,37]. As we have shown in this study and previous ones, the main advantage of bi-specific biological drugs over existing biological drugs, that comprise only one target, is a significant synergistic effect which cannot be obtained by simply administrating the functional activity units alone or in combination [22]. These synergistic effects have been mainly suggested to stem from the ability of bi-functional molecules to influence two or more biological pathways concomitantly [38]. Notably, the efficient apoptotic activity induced by CTLA4-FasL is highly specific for human B cell lymphoma cells that express both a functional Fas receptor and B7 receptors, supporting the notion that more than one biological signaling pathway are involved. Indeed, in B7 expressing cells, CTLA4-FasL provoked activation of the caspases cascade and abrogated anti-apoptotic signals at very low concentrations, a phenomena that could not be mimicked by CTLA4-Fc, sFasL or their combination. Most interestingly, abolishment of the c-IAP2 protein expression was seen only when B7-expressing cells were incubated with CTLA4-FasL and not with sFasL, even when the later was used at much higher concentrations, suggesting that effective Fas activation is not solely responsible for the effect observed, and that the CTLA4:B7 interaction of the fusion protein might play a separate significant role. Of note, cIAP and RIP have been implicated before as responsible for some tumors’ resistance to FasL or TRAIL mediated apoptosis [39,40], and c-IAP antagonists have been shown to sensitize cancer cells to TRAIL-induced apoptosis [41]. Significantly, in B7 negative cells this dual effect of CTLA4-FasL could not be elicited, though at higher concentration of CTLA4-FasL, effective activation of the casapses was observed. Importantly, this also suggests that measuring the expression of Fas, CD80 and CD86 in patient tumor samples may be used as a biomarker for patient that might benefit from this treatment.

Intriguingly, CTLA4-FasL potency was higher than that of trimeric FasL, CTLA4-Fc or their combination even when incubated with non-B7 expressing cells, making other explanations for its robust potency plausible. In this study we present data suggesting that a hexameric, higher-order CTLA4-FasL structures may play a significant role in the activity and potency of these novel bi-specific drugs, as has been shown for FasL [15,23].

As reported for other TNF-super family members, activation of the Fas apoptosis pathway requires trimerization of Fas receptors upon binding of FasL trimers [12]. Moreover, it was previously shown that efficient Fas activation requires two adjacent such trimerization events [15] and that hexameric forms of FasL are highly effective in apoptosis induction [19]. Therefore, the finding that the natural stochiometry of soluble CTLA4-FasL is a homo-hexamer is of great significance for understanding its unique, robust apoptotic capabilities. Being a hexamer, CTLA4-FasL is capable of presenting two functional trimers of FasL to their relevant receptors, resulting in optimal initiation of the apoptosis signaling pathway to the malignant cells.

**Figure 8:**
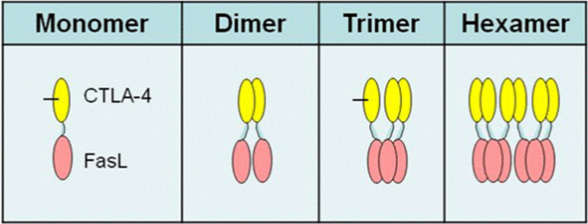
**Schematic model of the CTLA4-FasL homo-hexamer.** Monomer represents one domain of the CTLA-4 connected to one domain of the FasL. Trimer is composed of the FasL trimer and three domains of the CTLA-4, while two of them are connected by a disulfige bridge. Two CTLA4-FasL trimers may form a homo-hexamer, made of dimer of trimmers or trimer of dimers, resulting in a shift towards a small fraction of a dodecamer state.

The formation of a membrane bound CTLA4-FasL homo-hexamer was suggested previously [19]. Since only homo-trimers were identified at that earlier study, the authors suggested that two CTLA4-FasL trimers may form a homo-hexamer on target cell’s surface when anchored to B7 molecules, thereby inducing an extremely efficient apoptotic effect that would explain the high efficacy of CTLA4-FasL observed in that report. Here we present data suggesting that CTLA4-FasL naturally form a soluble and stable homo-hexamer as early as it is produced and that this structure maintains its stability through a purification process that includes harsh conditions and multiple freeze/though cycles (not shown). The stable hexameric structure can be explained by the fact that CTLA4 naturally forms a disulfide-linked dimer, while FasL naturally forms a stable trimer, thus, as suggested in Figure 8, a CTLA4-FasL trimer would possess an “open cysteine” that could link one such trimer to a second trimer, forming a stable CTLA4-FasL homo-hexamer.

Using a xenograft human-mouse disease model we show that CTLA4-FasL has the ability to inhibit the growth of tumors originating from B lymphocytes lineage, and to provide a significant beneficial effect on mice survival, in a dose dependent manner and at very low dosages. We show that this *in-vivo* effect is mediated by activation of the caspases cascade, as can be seen by the increased cleaved caspase 3 in immunohistichemistry of the tumors.

## Conclusions

In summary, in this study we present data that the fusion protein, CTLA4-FasL induces effective apoptosis of B lymphoblastoid cells, *in-vitro* and *in-vivo*, in a highly efficient way. Also, in the case of B7 expressing cells, its potency stems from the combination of its synergistic effect of activating the caspases cascade while abrogating the anti-apoptotic signaling, with its unique natural hexameric structure. We believe that this combination of properties, make CTLA4-FasL an extremely potent apoptosis inducer of B7 expressing tumors, such as B cell lymphomas.

## Materials and methods

### Protein production and purification

**Figure 9:**
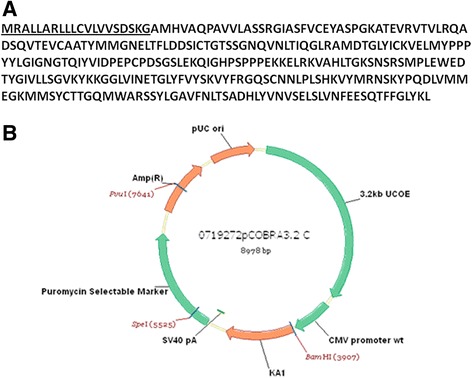
**CTLA4-FasL amino-acid sequence. (A)** The amino-acid sequence of the CTLA4-FasL. The underlined sequence represents the signal peptide of the human Urokinase protein, utilized to secrete the protein out of the cell. **(B)** A schematic map of the CTLA4-FasL cloned in UCOE expression plasmid vector.

The DNA encoding for CTLA4-FasL was synthesized at GENEART (Germany) based on the amino-acid sequence indicated in Figure 9A, and cloned into a UCOE expression vector (Cobra Biologics, Figure 9B).

CHO-S cells (Life technologies GIBCO, Invitrogen Corporation, NY, USA) were grown in CD-CHO medium (Life technologies) and transfected with 30 micrograms linearised DNA using DMRIE-C (Life technologies). Puromycin (Invitrogen) at 12.5 micrograms/ml was used for selection. CTLA4-FasL in culture media was quantified by a commercial FasL ELISA kit (e-Bioscience, CA, USA). Clones with the highest expression were expanded. One clone, with highest level of expression, was selected for limiting dilution, after which a final clone was selected based on growth profile analysis and CTLA4-FasL expression levels, tested by ELISA.

The selected clone was inoculated into a 50 L single use bioreactor. Cultivation and fed batch process medium was 50% CD CHO (Invitrogen), 50% EX-CELL® CHO 5 (SAFC, SIGMA-ALDRICH), supplemented with 8 mM Glutamax and 1× HT (Hypoxathine 0.1 mM, Thymidine 0.016 mM) (Invitrogen). The titer of CTLA4-FasL at time of harvest was 50 mg/L (Gyrolab platform immunoassay; see below).

To purify the protein, thawed production harvest was centrifuged at 5000 g, followed by 0.2 μm filtration (10 kDa cut-off cellulose centrifugal filters) (Sartorius-Stedim, Goettingen, Germany) and loaded onto a Concavalin-A (Con-A) HiTrap column (GE Healthcare, Little Chalfont, UK) at 7 mg/mL resin. The Con-A eluate loaded onto a Size-Exclusion-Chromatography (SEC) Sephacryl S-200 column (GE Healthcare). The SEC eluate was 0.2 μm filtered (Minisart syringe filter) (Sartorius-Stedim) and frozen at -70°C.

### His6-tagged protein

Some of the *in-vitro* experiments were performed with a His_6_ tagged version of CTLA4-FasL [42]. The activity of the tagged His_6_CTLA4-FasL was compared to that of the purified non-tagged CTLA4-FasL and found to be identical (not shown).

### Cell lines

Liver adenocarcinoma Sk-Hep1 cell line [43], A498 Renal Carcinoma Cell line [44] and Raji B cell lymphoma cell line [45] were purchased from ATCC (Manassas, Virginia, USA). The JY lymphoblastoid cell line [22] was a kind gift from Prof. M.L. Tykocinski laboratory, Jefferson Medical School, PA, USA. Other cell lines were a kind gift from the Gene Therapy institute and Hepatology Unit, Hadassah Hebrew University Medical Center in Jerusalem, Israel. Attached cells were grown in DMEM (Gibco) supplemented with 10% FBS, 2 mM glutamine, 100 IU/mL penicillin and 100 μg/mL streptomycin, and were detached using Trypsin-EDTA solution. Suspended cells were grown in RPMI (Gibco) with the same additives. All cell lines were cultured at 37°C, 6% CO_2_, and tested periodically for mycoplasma contamination using EZ-PCR mycoplasma test kit (Biological Industries, Israel).

### Activity bioassay

For *in-vitro* examination of the CTLA4-FasL cytotoxic effect on different human cell lines, 32,000 cells per well (suspended cultures) or 8000 cells per well (attached cells) in 50 ul of complete RPMI (suspended cultures) or DMEM (attached cells) medium without Phenol Red, were seeded in triplicates, in a flat 96-wells plate (Nunc or similar), and 50 ul of CTLA-4-FasL (or his_6_CTLA-4-FasL) dilutions (in growth media; 3000 ng/ml-0.1 ng/ml, triplicates), or dilution media as negative control were added. Calibration curve wells contained serial dilution from 64,000 to 2000 cells per well for suspended cultures or 16,000 to-2000 cells for attached cells in triplicates. Plates were incubated for 24 hours at 37°C in 6% CO_2_ humidified incubator. Cell viability was quantified by a MTS kit (Promega, CellTiter 96® Aqueous Non-Radioactive Cell Proliferation Assay) according to manufacturer instructions.

### SDS-PAGE, western blot and native-PAGE analysis

For CTLA4-FasL and CD40-FasL SDS-PAGE and western blots, 4-12% Bis-Tris gel (1 mm, 12 wells, NP0322BOX, Life Technologies) and “See Blue Plus 2” MW markers (LC5925, Life Technologies) were used. After blocking (skim milk) membranes (PVDF) were incubated with either goat anti-human CTLA4 antibody (AF-386-PB, R&D Systems, 1:300 dilution) or goat anti-human Fas Ligand (AB126, R&D Systems, 1:100 dilution). The secondary antibody was a donkey anti-Sheep/Goat Immunoglobulins (HRP, AP360, The Binding Site, 1:10,000 dilution), detected by HRP substrate 3,3′, 5,5′ – Tetramethylbenzidine (TMB, Liquid Substrate System for Membranes, Sigma-Aldrich, MO, USA).

For western blot analysis of intracellular proteins, whole cell lysate were separated on 12% SDS-PAGE and blotted according to standard procedures. Membranes were incubated with the following primary antibodies: anti Caspase-3, Caspase-8, Caspase-9, PARP, Bcl-2, c-IAP-1, c-IAP2, RIP all from Cell Signaling Technology, Danvers, MA, USA; anti XIAP (Santa Cruz Biotechnology, Santa Cruz, CA, USA); anti FLIP (Enzo, CA, USA); anti BID, anti GAPDH (Millipore, Billerica, MA, USA); anti IkB-α (R&D). Secondary detection was performed with HRP-conjugated antibodies (BioRad, Hercules, CA, USA). In some experiments blocking anti CD80 and/or CD86 Abs (MAB140 and MAB141 respectively, R&D, USA) were added to the culture.

Native-PAGE analysis was performed with NativePAGE™ Novex® 4-16% Bis-Tris Gel (Invitrogen), according to the manufacturer protocol. Samples were prepared with and without G-250 sample additive. 10uL of the CTLA4-FasL sample and of the NativeMark were loaded to each gel lane. Coomassie G-250 (Invitrogen) was added to the cathode buffer and to the samples, resulting in staining of the proteins during gel electrophoresis.

### Gyrolab

To efficiently quantify CTLA4-FasL, a Gyrolab platform immunoassay (Gyrolab Workstation, Gyros, Uppsala, Sweden) was developed. An anti-Human CTLA-4 polyclonal goat antibody (AF-386 PB, R&D systems) was selected as capture antibody, and was biotinylated using EZ-link Sulfo-NHS-LC-Biotin (PIERCE, Thermo Fisher Scientific Inc, IL, USA) according to manufactures protocol. An anti-Human Fas Ligand monoclonal mouse IgG2B antibody (MAB-126, R&D Systems) was selected as detection antibody and was Alexa labeled using the Alexa Fluor™ 647 Monoclonal Antibody Labelling Kit (Molecular probes, Life technologies) according to manufacture's protocol.

CTLA4-FasL sample and standards were diluted into the range 0.1 – 100 μg/ml using Rexxip CSS (Gyros, Sweden) before being applied onto a CD Bioaffy 20 HC microlaboratory disc (Gyros). CTLA4-FasL sample (20 nl) were transferred by centrifugal force through minute columns (15 ml) packed with Streptavidin beads to which biotinylated anti-CTLA4 antibodies were attached. Detection of CTLA4-FasL bound to the column was performed after addition of Alexa labelled anti-FasL antibodies by laser induced fluorescence. Quantitation was performed relative to a 5 parameter logistic standard curve.

### CD40-FasL – production and ELISA

CD40-FasL was produced as described before [46]. For quantification CD40 Human ELISA Kit (Abcam, UK) was used according to manufacturer’s instructions.

### FACS analysis

1 × 10^6^ cells were washed with PBS and re-suspended in 95 μl of staining buffer (1% BSA, 0.1% azide in PBS) and 5 μl of human Fc blocker (e-Bioscience), and incubated on ice for 5′. Cells were immunostained with PE-anti hCD95 (eBioscience), APC-anti hCD86 (BD) or FITC-anti hCD80 (BD) or matching isotype Abs (PE-mouse IgG1 kappa, APC-mouse IgG1 kappa or FITC-mouse IgG1 kappa, respectively, all from eBioscience) on ice for 30′ and 20,000 events per sample were counted using a BD™ LSR II Flow Cytometer, and data were analyzed using CellQuest software (Becton Dickinson).

### Size-exclusion - HPLC

Analytical size-exclusion (SE) was performed using a Dionex HPLC instrument (Pump P580, Auto sampler ASI-100/ASI-100 T Injector, UV/VIS Detector UVD340U, Chromeleon 6.80 Software) with Tosoh Bioscience TSK-Gel G3000SWXL 7.8×300 mm column. Phosphate Buffered Saline (PBS) was used as the mobile phase and samples of <50 μg or 100 μg were injected.

Reference standards and 25% Gel Filtration Standard (GFS, BioRad) were run before and after the samples. The separation was performed using an isocratic separation method with a runtime of 20 min and a flow rate of 1 ml/min. The column oven was set at 25°C and the sample holder at 8°C. The size distribution profiles were recorded using UV absorption at 214 nm.

### Iso Electric Focusing (IEF)

CTLA4-FasL was separated on IEF gels (Novex, Life technologies, NY, USA), at pH3-7 and pH3-10, according to manufacturer instructions.

### Gel filtration chromatography

Protein (1 mg/ml; 400 ul) was applied to a Superdex 75 analytical column (30 × 1 cm; GE-Healthcare) using AKTA Explorer (GE-Healthcare) and eluted at a flow rate of 0.8 ml/min in buffer PBS monitoring absorbance at 280, 260 and 220 nm. Molecular weight standards catalase (232 kDa), aldolase (163 kDa), BSA (67 kDa), OvoAlbumin (44 kDa), Chymotrypsinogen A (25 kDa) and RNaseA (13.7 kDa) (GE-Healthcare) were used. For CD40-FasL containing medium the molecular weight standards are as presented in Additional file 2: Figure S2A.

### Xenograft lymphoma model

Athymic-NUDE female mice (Harlan, Israel), 4-6 weeks of age, were maintained under defined flora conditions at the Hebrew University Pathogen-Free Animal Facility. All experiments were approved by the Animal Care Committee of the Hebrew University. The JY cells used in this study were harvested from subcutaneous JY xenograft tumor, and expanded in culture. Mice were irradiated (300R), and two days later JY cells in exponential growth were harvested, washed with PBS, and injected subcutaneously (7-10 × 10^6^/mouse) into the right flanks of mice. Treatment was started to each mouse individually when tumor was palpable and could be measured, between day 4 to 7 after cells injection (most of the animals were treated from day 5).

Mice were treated for 4 days with two 100 μl subcutaneous injections per day of CTLA4-FasL or the vehicle buffer (PBS). Tumor size was measured by a micro caliber and volume was calculated by the equation: (w^2^*length/2). Mice bearing tumor of >1000 mm^3^ or necrotic tumors were sacrificed. In some experiments, and to further assess CTLA4-FasL effect on JY-derived tumors, mice were sacrificed one hour post the 1st injection, at the 4^th^ injection day (20 μg CTLA4-FasL per day). For livers histology examination animals treated with vehicle or CTLA4-FasL (100 mcg/day, 4 days) were sacrificed 8-30 days after the last injection, and livers harvested and fixated in 4% formaldehyde, routinely processed, and embedded in paraffin. Transverse sections (5 μm) were stained with hematoxylin and eosin (H&E).

### Pharmacokinetics

For analysis of pharmacokinetics, CTLA4-FasL at different doses was subcutaneously injected to mice at a total volume of 150 μl per mouse. Mice were sacrificed at various time points post injection. Blood was collected in heparin, kept on ice, centrifuged at 1000 g (~3000 rpm) for 10′, plasma was kept at -70°C. CTLA4-FasL was quantified by LEGEND MAXTM Human Soluble CTLA-4 ELISA kit (#437407, Biolegend, CA, USA), according to the manufacturer's instructions.

### Immunohistochemistry

To assess CTLA4-FasL apoptosis-inducing effect *in-vivo*, Athymic-NUDE mice bearing JY tumors were sacrificed one hour post 10 ug injection, at the 5th injection day (20 ug per day, sc, divided to two daily 10ug injections, 4 hours apart). Subcutaneous tumors were removed, fixated in 4% formaldehyde, and embedded in paraffin. Sections (5 μm) were deparaffinized in xylene (3×3′) and rehydrated in graded alcohol (3×1′ 100% ethanol; 3×1′ 96% ethanol). Following 5′ incubation in 3% H_2_O_2_ for endogenous peroxidase inactivation, slides were incubated in Citrate buffer (pH6; Invitrogen) and boiled in electric pressure cooker (BioCare Medical, CA, USA) for antigen retrieval. Samples were blocked for 30′ in CAS-BLOCK (Invitrogen) prior to overnight incubation with the anti-cleaved caspase 3 primary antibody (Cell Signaling #9661; 1:100 diluted in CAS-BLOCK) at 4°C in humidified box. Following washing (3×2′ in Super Sensitive wash buffer, BioGenex), samples were incubated for 30′ at RT with the Simple stain MAX PO (MULTI) anti-rabbit immune-peroxidase polymer (NICHIREI BIOSCIENCES INC.). Diaminobenzidine (DAB; UltraVision Detection System, Thermo scientific, MA, USA) was used as the chromogen according to manufacturer's instructions, and 20'' incubation in hematoxylene (SIGMA-Aldrich) was used as the nuclear counter-stain. Following dehydration steps (2′ 80% ethanol, 2′ 96% ethanol, 2′ 100% ethanol, 2′ xylene) and mounting (Histomount mounting solution, Invitrogen), ×20 pictures were taken by the Nikon ECLIPSE Ti light microscope and captured by the DSFI-1 camera (Nikon, USA).

## Additional files

Additional file 1: Figure S1.
*A CTLA4-FasL effects on JY – B lymphoblastic cell line viability.* 32,000 cells per well were incubated in the presence or absence of CTLA-4-FasL (3000ng/ml-0.1ng/ml, triplicates) for 24 hours. Cell viability was quantified by a MTS kit. B. Expression of CD80, CD86 and CD95 on Raji cells surface. Raji cells were immunostained with PE-anti hCD95, APC-anti hCD86 or FITC-anti hCD80 or matching isotype Abs 20,000 events per sample were counted using a BD™ LSR II Flow Cytometer. Dot plots are presented. Data were analyzed using CellQuest software (Becton Dickinson). Additional file 2: Figure S2.
*Gel filtration analysis of CD40-FasL indicating a size compatible with a homohexamer.* CD40-FasL containing media samples were fractionized in high resolution gel filtration Superdex 200 size exclusion chromatography. Collected fractions were analyzed by Western blot and ELISA. A. Molecular weight standards for the gel filtration fractions. B. Western blots of fractions 4-11 from the gel filtration of the CD40-FasL containing media using anti-FasL Abs. C. ELISA detecting human CD40 of fractions 5-14 from the gel filtration of the CD40-FasL containing media. Additional file 3: Figure S3.
*CTLA4-FasL effects on the expression of pro-apoptotic and anti-apoptotic proteins in B cell lymphoma (1 A-G) and RCC (H) cell lines*. Raji and A498 cell lines were incubated with indicated concentrations of CTLA4-FasL, sFasL, CTLA4-Ig or the combination of the later two for 2h at 37°C. Whole cell lysates were analyzed by Western blot using the indicated Abs. Data was normalized against GAPDH by a Quantity One program (BioRad, Version 4.6.9). Data are presented as mean ± SE. P<0.05; CTLA4-FasL a: vs Untreated (UT), b: vs sFasL, c: vs CTLA-4-Ig, d: vs sFasL + CTLA-4-Ig.
