# Supplementary figures and images for: Highly efficient, In-vivo Fas-mediated Apoptosis of B-cell Lymphoma by Hexameric CTLA4-FasL
Source: J Hematol Oncol. 2014 Sep 17;7:64. doi: 10.1186/s13045-014-0064-6 (PMC4189725; doi:10.1186/s13045-014-0064-6)

## Slide 1
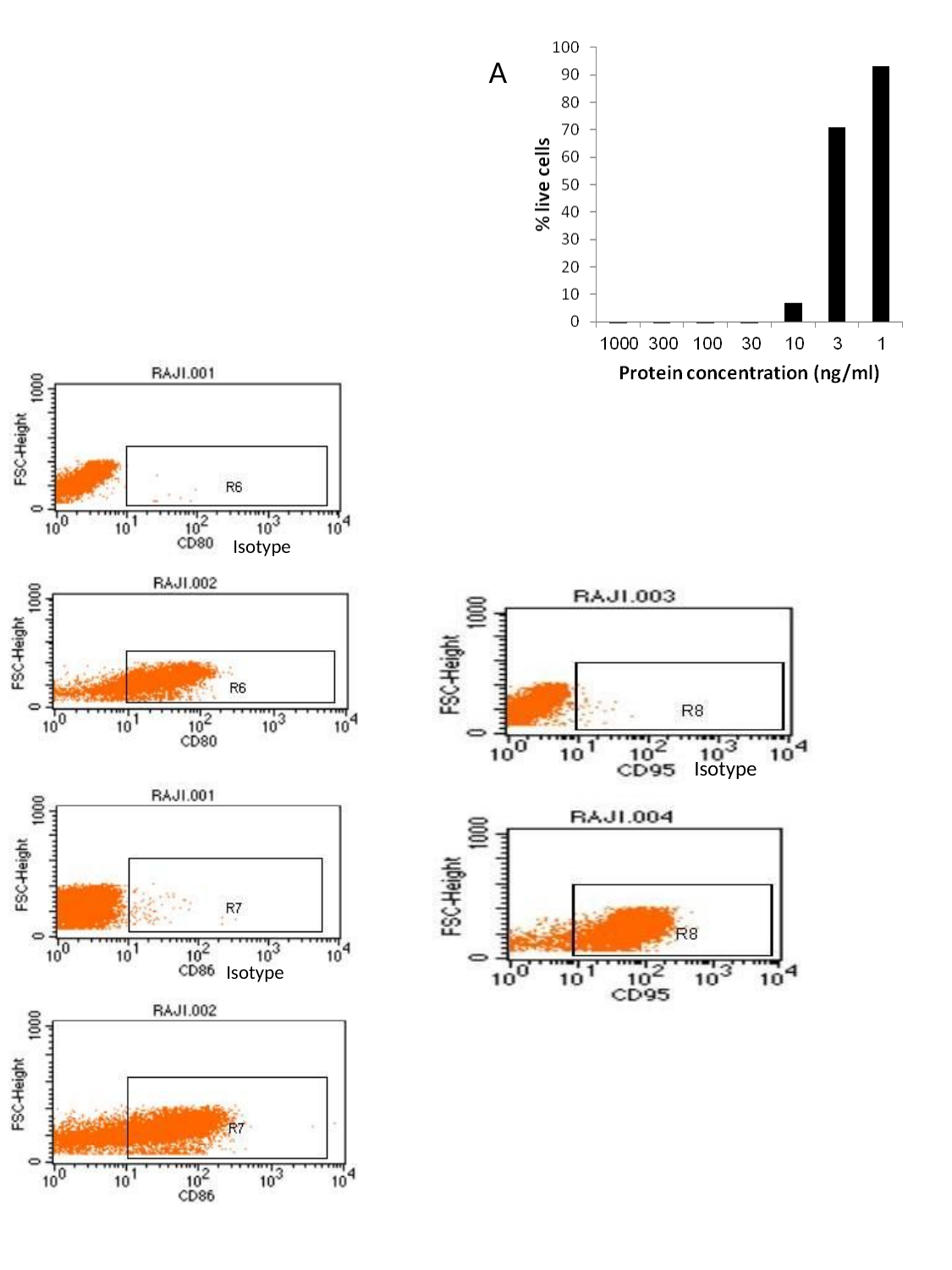

Isotype
Isotype
Isotype
A
B

Supplement: Additional file 1: Figure S1. — A CTLA4-FasL effects on JY – B lymphoblastic cell line viability. 32,000 cells per well were incubated in the presence or absence of CTLA-4-FasL (3000ng/ml-0.1ng/ml, triplicates) for 24 hours. Cell viability was quantified by a MTS kit. B. Expression of CD80, CD86 and CD95 on Raji cells surface. Raji cells were immunostained with PE-anti hCD95, APC-anti hCD86 or FITC-anti hCD80 or matching isotype Abs 20,000 events per sample were counted using a BD™ LSR II Flow Cytometer. Dot plots are presented. Data were analyzed using CellQuest software (Becton Dickinson). [file 13045_2014_64_MOESM1_ESM.ppt]

## Slide 1
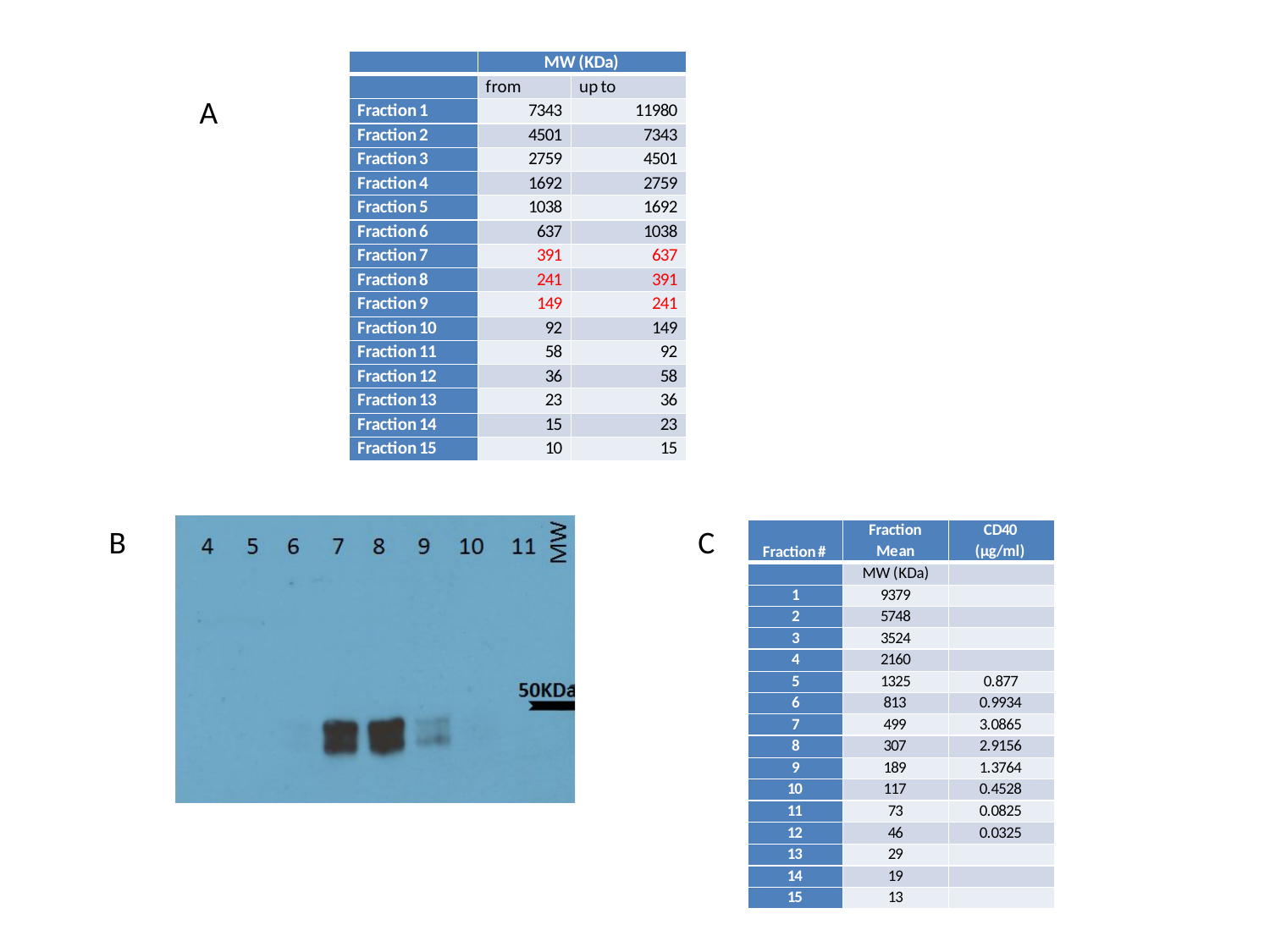

A
B
C

Supplement: Additional file 2: Figure S2. — Gel filtration analysis of CD40-FasL indicating a size compatible with a homohexamer. CD40-FasL containing media samples were fractionized in high resolution gel filtration Superdex 200 size exclusion chromatography. Collected fractions were analyzed by Western blot and ELISA. A. Molecular weight standards for the gel filtration fractions. B. Western blots of fractions 4-11 from the gel filtration of the CD40-FasL containing media using anti-FasL Abs. C. ELISA detecting human CD40 of fractions 5-14 from the gel filtration of the CD40-FasL containing media. [file 13045_2014_64_MOESM2_ESM.ppt]
